# Supplementary figures and images for: Lower dormancy with rapid germination is an important strategy for seeds in an arid zone with unpredictable rainfall
Source: PLoS One. 2019 Sep 10;14(9):e0218421. doi: 10.1371/journal.pone.0218421 (PMC6736279; doi:10.1371/journal.pone.0218421)

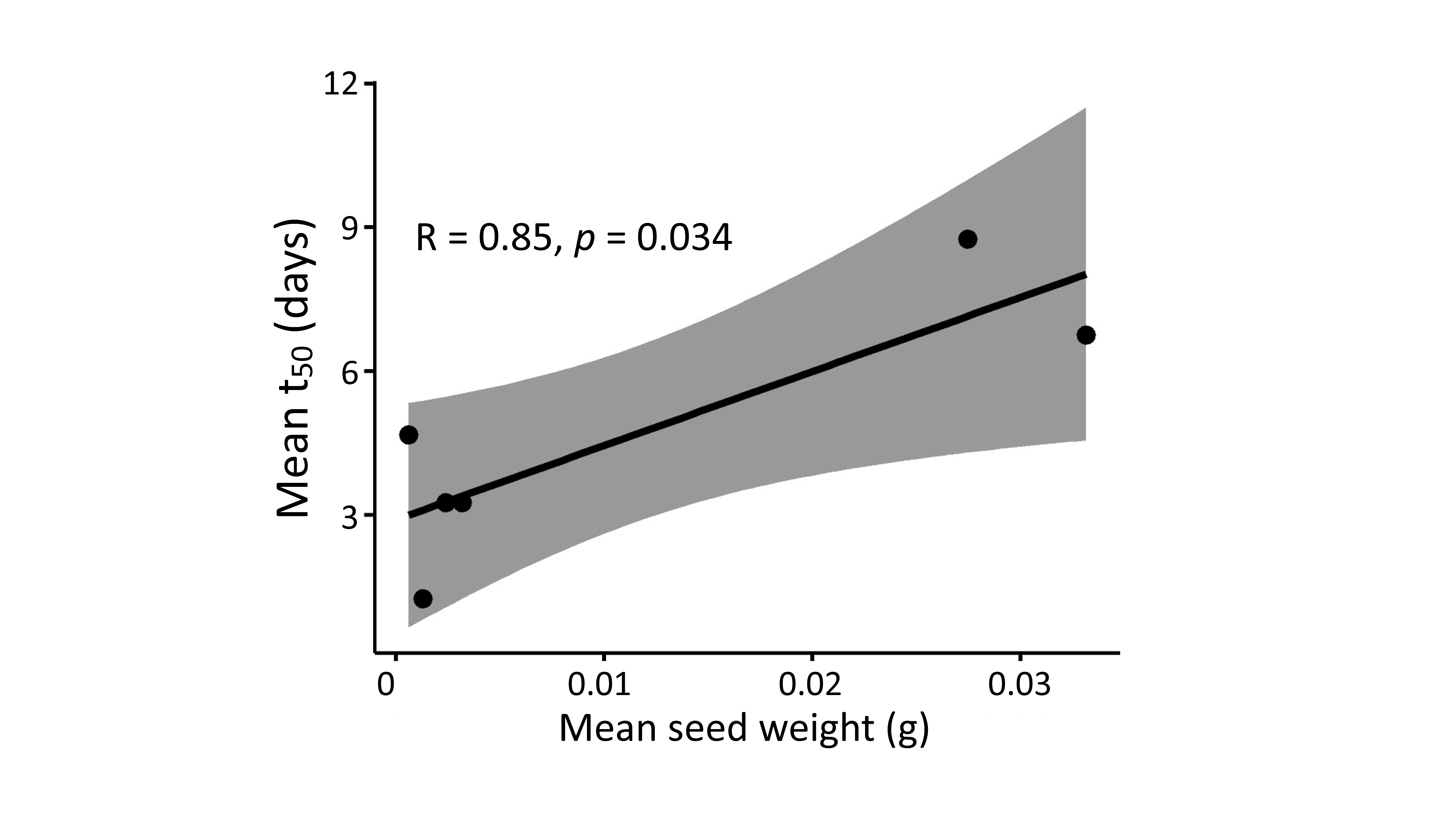

Supplement: S1 Fig — (TIF) [file pone.0218421.s001.tif]
