# Supplementary material for: Lower dormancy with rapid germination is an important strategy for seeds in an arid zone with unpredictable rainfall
Source: PLoS One. 2019 Sep 10;14(9):e0218421. doi: 10.1371/journal.pone.0218421 (PMC6736279; doi:10.1371/journal.pone.0218421)
Supplement: S1 Table — Maximum germination is also shown for the GA3 treatment (when diurnal temperature is 30/20°C), and after the after-ripening treatment for A. rhagodioides. Letters indicate the results of Tukey pairwise comparisons among the three diurnal temperature treatments. Treatments that share a letter are not significantly different from each other. For the GA3 and after-ripening treatments, asterisks represent significant differences compared to the control treatment. For the ‘GA3 + after-ripening’ treatment, the comparison is to the GA3 only treatment (n.s. = not significant; * 0.05 > p > 0.01; ** 0.01 > p > 0.001; p < 0.001). (DOCX) [file pone.0218421.s002.docx]

**S1 Table. Mean values for maximum germination for each species under three diurnal temperatures.**

| **Treatments** | *Atriplex rhagodioides* | *Maireana sedifolia* | *Maireana pyramidata* | *Casuarina pauper* | *Hakea tephrosperma* | *Alectryon oleifolius* |
| --- | --- | --- | --- | --- | --- | --- |
| *Diurnal temp* | | | | | | |
| 30/20 | 50.8 (6.8)^a^ | 95.7 (3.7)^a^ | 66.7 (2.7)^a^ | 94.3 (5.5)^a^ | 92.7 (2)^a^ | 80 (7.7)^a^ |
| 25/15 | 69.2 (2.9)^b^ | 100 (1.1)^a^ | 86.7 (4.7)^b^ | 100 (9.8)^a^ | 100 (1.7)^b^ | 76.7 (11.4)^a^ |
| 17/7 | 60 (2.9)^ab^ | 97.8 (2.7)^a^ | 88.3 (4.2)^b^ | 77.1 (9.8)^a^ | 100 (1.7)^b^ | 66.7 (5.4)^a^ |
| *GA_3_* |  |  |  |  |  |  |
| 30/20 | 86.2 (6.6)** | 98.9 (1.8)^n.s.^ | 63.3 (4.3)^n.s.^ | 97.1 (7.4)^n.s.^ | 96.9 (2)^n.s.^ | 86.7 (8.6)^n.s.^ |
| *After ripening* | | | | | | |
| 30/20 | 94.9 (4.9)** |  |  |  |  |  |
| 25/15 | 93.5 (6.2)* |  |  |  |  |  |
| 17/7 | 98.9 (3.6)*** |  |  |  |  |  |
| *GA_3_ + after ripening* | | | | | | |
| 30/20 | 93.5 (2.3)^n.s.^ |  |  |  |  |  |

Maximum germination is also shown for the GA_3_ treatment (when diurnal temperature is 30/20°C), and after the after-ripening treatment for *A. rhagodioides*. Letters indicate the results of Tukey pairwise comparisons among the three diurnal temperature treatments. Treatments that share a letter are not significantly different from each other. For the GA_3_ and after-ripening treatments, asterisks represent significant differences compared to the control treatment. For the ‘GA_3_ + after-ripening’ treatment, the comparison is to the GA_3_ only treatment (n.s. = not significant; * 0.05 > p > 0.01; ** 0.01 > p > 0.001; p < 0.001).
