# Supplementary material for: Lower dormancy with rapid germination is an important strategy for seeds in an arid zone with unpredictable rainfall
Source: PLoS One. 2019 Sep 10;14(9):e0218421. doi: 10.1371/journal.pone.0218421 (PMC6736279; doi:10.1371/journal.pone.0218421)
Supplement: S2 Table — Atriplex rhagodioides (AR) refer to seeds rendered non-dormant through a 12 month after-ripening. (DOCX) [file pone.0218421.s003.docx]

**S2 Table. Time to minimum, 50% and maximum germination of seeds** incubated at 30/20°C, 25/15°C and 17/7°C (± standard error). *Atriplex rhagodioides* (AR) refer to seeds rendered non-dormant through a 12 month after-ripening.

|  | **Mean t_min_** | | | | **Mean t_50_** | | | | **Mean t_max_** | | |
| --- | --- | --- | --- | --- | --- | --- | --- | --- | --- | --- | --- |
| **Species** | 17/7°C | 25/15°C | 30/20°C | 17/7°C | | 25/15°C | 30/20°C | 17/7°C | | 25/15°C | 30/20°C |
| *Alectryon oleifolius* | 16.0 (0.9) | 6.5 (0.5) | 5.6 (0.5) | 20.3 (0.9) | | 12.3 (2.7) | 8.8 (1.7) | 21.8 (1.0) | | 14.8 (1.8) | 10.3 (1.3) |
| *Casuarina pauper* | 6.5 (0.3) | 3.3 (0.3) | 2.0 (0.0) | 9.0 (0.6) | | 4.5 (1.2) | 3.3 (0.3) | 9.8 (0.6) | | 7.5 (1.0) | 7.5 (1.04) |
| *Hakea tephrosperma* | 7.8 (0.3) | 4.8 (0.3) | 4.3 (0.5) | 10.0 (0.0) | | 7.3 (0.3) | 6.8 (0.3) | 11.3 (0.3) | | 9.3 (0.8) | 8.5 (0.5) |
| *Atriplex rhagodioides* | 14.0 (1.8) | 8.3 (0.9) | 7.8 (0.9) | 24.8 (1.0) | | 18.5 (1.0) | 16.5 (1.8) | 26.0 (0.4) | | 21.8 (0.3) | 20.0 (0.6) |
| *Atriplex rhagodioides* (AR) | 6.0 (0.0) | 3.0 (0.0) | 3.0 (0.0) | 8.7 (0.3) | | 5.0 (0.0) | 4.7 (0.3) | 12.7 (0.3) | | 9.7 (0.3) | 9.0 (0.0) |
| *Maireana pyramidata* | 1.5 (0.3) | 1.0 (0.0) | 1.0 (0.0) | 3.8 (0.3) | | 2.5 (0.29) | 3.3 (0.3) | 5.0 (0.0) | | 4.0 (0.0) | 3.5 (0.3) |
| *Maireana. sedifolia* | 1.0 (0.0) | 1.0 (0.0) | 1.0 (0.0) | 2.3 (0.3) | | 1.3 (0.3) | 1.3 (0.3) | 4.0 (0.0) | | 2.8 (0.3) | 2.0 (0.0) |
